# Surface display of glycosylated Tyrosinase related protein-2 (TRP-2) tumour antigen on *Lactococcus lactis*

**DOI:** 10.1186/s12896-015-0231-z

**Published:** 2015-12-29

**Authors:** Jeevanathan Kalyanasundram, Suet Lin Chia, Adelene Ai-Lian Song, Abdul Rahim Raha, Howard A. Young, Khatijah Yusoff

**Affiliations:** Department of Cell and Molecular Biology, Faculty of Biotechnology and Biomolecular Sciences, Universiti Putra Malaysia, Serdang, Selangor Darul Ehsan Malaysia; Department of Microbiology, Faculty of Biotechnology and Biomolecular Sciences, Universiti Putra Malaysia, 43400 UPM Serdang, Selangor Darul Ehsan Malaysia; Institute of Bioscience, Universiti Putra Malaysia, Serdang, Selangor Darul Ehsan Malaysia; Cancer and Inflammation Program, Center for Cancer Research, National Cancer Institute, Frederick, MD USA

**Keywords:** Surface display, *Lactococcus lactis*, Lysin motif, Tyrosinase related protein-2

## Abstract

**Background:**

The exploitation of the surface display system of food and commensal lactic acid bacteria (LAB) for bacterial, viral, or protozoan antigen delivery has received strong interest recently. The Generally Regarded as Safe (GRAS) status of the *Lactococcus lactis* coupled with a non-recombinant strategy of *in-trans* surface display, provide a safe platform for therapeutic drug and vaccine development. However, production of therapeutic proteins fused with cell-wall anchoring motifs is predominantly limited to prokaryotic expression systems. This presents a major disadvantage in the surface display system particularly when glycosylation has been recently identified to significantly enhance epitope presentation. In this study, the glycosylated murine Tyrosinase related protein-2 (TRP-2) with the ability to anchor onto the *L. lactis* cell wall was produced in suspension adapted Chinese Hamster Ovary (CHO-S) cells by expressing TRP-2 fused with cell wall anchoring LysM motif (cA) at the C-terminus.

**Results:**

A total amount of 33 μg of partially purified TRP-2-cA from ~6.0 g in wet weight of CHO-S cells was purified by His-tag affinity chromatography. The purified TRP-2-cA protein was shown to be N-glycosylated and successfully anchored to the *L. lactis* cell wall.

**Conclusions:**

Thus cell surface presentation of glycosylated mammalian antigens may now permit development of novel and inexpensive vaccine platforms.

## Background

Surface display of engineered antigens on the Lactic acid bacteria (LAB) is currently being investigated as promising cellular vehicles for vaccine delivery [1–3]. This approach manipulates the natural cell surface molecular localisation of proteins that direct fundamental biological processes such as cell to inter-cell recognition, signal transduction, surface anchoring, colonisation and immunological interaction in living organisms [4]. Besides their GRAS (Generally Regarded as Safe) status compared to their attenuated pathogenic counterparts, the LAB, with probiotic and immunomodulatory properties have the ability to survive passage through both animals and humans for up to 5–7 days [5]. This has made LAB an excellent candidate for oral and intranasal vaccine development [6, 7]. Therefore, LAB such as *Lactococcus lactis* can be genetically engineered to become an efficient recombinant cell factory for DNA delivery as well as production and presentation of antigens [6, 8]. The presentation of antigens through surface display or secretion by *L. lactis* in numerous studies utilises the well understood and characterised surface binding protein domain such as transmembrane domains, lysin motif (LysM) and LPXTG motifs [9, 10].

Based on the findings described above, the LAB have the potential to be developed as a tumour antigen carrier for therapeutic or prophylactic cancer vaccines. Such cancer vaccines would be able to mount sustainable immune responses to eradicate primary tumours as well as prevent cancer relapses [11]. Since the early discovery of probiotic anti-tumour activity [12], the LAB have been primarily manipulated as prophylactic adjuvants for prevention of colorectal cancer [13] as well as breast and bladder cancers, albeit to a lesser extent. However, the exact mechanism of LAB anti-tumour activity has not been fully understood. It has been hypothesised that the competitive inhibition of LAB in the intestinal microflora may have resulted in disruption of metabolic equilibrium, digestion of potential carcinogens and promotion of a T helper 1 immune response through mucosal immunity [14, 15]. Cancer antigen delivery by the LAB, on the other hand, has not been widely explored and is only limited to two examples: 1) surface display of viral antigens from the human papillomavirus type-16 (HPV-16) E7 antigen on *L. lactis*, *Lactobacillus plantarum* and *Lactobacillus casei* for cervical cancer treatment [6, 16] as well as 2) oncofetal antigen surface display by *L. plantarum* [17].

TRP-2 (Tyrosinase related protein-2) is an enzyme involved in melanin synthesis which undergoes N-glycosylation and translocates into the melanosome in melanocytes. It has been reported to be a tumour-associated antigen present in both melanocytes and melanoma and as such, TRP-2 has been intensely studied as a viable therapeutic and prophylactic vaccine candidate for melanoma and glioblastoma [18, 19]. The TRP-2 DNA vaccination for glioblastoma multiforme treatment has resulted in tumour regression and immunological targeting to increase chemotherapeutic drug sensitivity [19, 20]. Therapeutic effects for melanoma by alphavirus (Venezuelan equine encephalitis virus, VEE) replicon [21], cytomegalovirus (CMV) [22], attenuated *Salmonella typhimurium* [23] and *Listeria monocytogenes* [24] carrying TRP-2 have also been reported. Surprisingly, despite good documentation of the role of LABs as adjuvants in mucosal immunogenicity [25, 26], these GRAS status bacteria have yet to be manipulated to express TRP-2 for both therapeutic and prophylactic settings. In addition, common autoimmunity side effects of hypopigmentation (vitiligo) resulting from TRP-2 (self-antigen) immunization have been observed to be dependent on the vaccine strategies [21, 27].

In this study, live *L. lactis* surface displaying post-translationally modified TRP-2 *in trans* was developed with the intention of future use as a prophylactic and therapeutic cancer vaccine. To date, bacterial surface display systems for heterologous protein mainly utilize a recombinant surface display mechanism. However, these recombinant bacterial surface display systems are very much limited to non-glycosylated proteins [2, 3, 27, 28] since prokaryotic cells, which in general do not undergo post-translational modifications, are used as cell factories to produce the recombinant proteins fused to the surface display anchor domain. Thus, the recombinant bacterial surface display system may not be suitable if glycosylation is vital for the folding and function of target proteins [29]. The recent emergence of the *in-trans* surface display system [9, 30, 31] provides an attractive platform for glycoprotein delivery. In the *in-trans* system, the protein of interest is fused with the target bacteria’s surface anchoring motif (SAM), which is expressed and purified from a separate host cell before it is externally introduced to the bacterial cell wall. This strategy was utilised in this study by producing TRP-2-cA glycoprotein fused with lactococcal cell wall anchor domain in Chinese Hamster Ovary (CHO) cells before subsequent binding to *L. lactis* cell wall. To our knowledge, this is the first report of prokaryotes surface displaying a glycosylated mammalian protein.

## Methods

### Bacterial strains and culture conditions

Plasmid construction and cloning was carried out by utilizing the TOP 10 *E. coli* strain (Invitrogen, USA). The TOP 10 *E. coli* (Invitrogen, USA) was grown in LB broth by incubating at 37 °C for 12-16 h and shaken at 250 rpm. During positive transformation screening and maintenance, the TOP 10 *E. coli* (Invitrogen, USA) strain was grown in LB agar/broth supplemented with 100 μg/ml of ampicillin.

### Gene amplification and plasmids construction

Both *trp-2*_1-472_ epitope and *cA* gene DNA template were codon optimized based on CHO cell codon frequency table (Codon Usage Database at http://www.kazusa.or.jp/codon/cgi-bin/showcodon.cgi?species=10029) in reference to *Mus musculus* cell-line B16F10 TRP-2 [Accession no: EU554632.1] and *L. lactis* N-acetylmuramidase LysM motifs [Accession no: U17696.1]. However the native murine TRP-2 signal peptide, *mtrp-2*_1-23_ was replaced with Chinese Hamster TRP-2, *Cricetulus griseus*, TRP-2, [Accession no: ERE88475.1] signal peptide, chTRP-2_1-23_ to promote target protein production in CHO-S cells. Additional linker sequence, 5’-GGCGGCTCCGGCGGCGGCTCCGGC-3’ which corresponds to GGSGGGSG amino acid linker sequence was incorporated upstream to the cA sequence for gene synthesis. Six histidine repeats with an enterokinase cleavage site were also incorporated downstream of the aforementioned codon optimized, synthesized cA sequence.

The *trp-2*_1-472_ epitope was amplified by *trp-2*_1-472_ forward primer, 5’-ATAT*AAGCTT*ACCATGGGCCTGGTGCACTG-3’ and reverse primer, 5’- ATAT*GGATCC*GTGGTGGTGGTGGTGGTGGCCGG -3’ containing *Hind*III and *BamH*I restriction sites respectively. The *cA* sequence was amplified by *cA* forward primer 5’- GCGC*GGATCC*GGCGGCTCCGGCGGCGGCTC-3’ and reverse primer 5’- ATAT*GCGGCCGC*TCAGTGGTGGTGGTGGTGGTGGCCGGTCC -3’ containing *BamH*I and *Not*I restriction sites. PCR reaction was carried out in 1× *Pfu* reaction buffer containing 2 mM MgSO_4_, 0.2 mM dNTP, 2 units of *Pfu* polymerase (Fermentas, USA), 0.5 μM of forward and reverse primers and approximately 20 ng of DNA template. The thermal cycler temperature condition for a 20 cycle amplification was set at a 94 °C /1 min, 62 °C/1 min and 72 °C /2.5 min with a final extension of 72 °C /10 min. Amplified *trp-2*_1-472_ and *cA* genes replicates were gel purified by using Wizard® SV Gel and PCR Clean-Up System (Promega, USA). Both of the purified genes were then digested with *BamH*I at 37 °C for 3 h separately and ligated using T4 DNA Ligase (Fermentas, USA) at 1:1 gene ratio overnight at 4 °C. The resulting DNA ligation mixture was used as a template to amplify *trp-2*_1-472_-*cA* fusion gene by using *trp-2*_1-472_ forward primer and *cA* reverse primer under abovementioned PCR buffer conditions and thermal cycler temperature profile. The *trp-2*_1-472_-*cA* fusion gene was gel purified as described previously. The pcDNA: *trp-2*_1-472_-*cA* expression plasmid was constructed by ligating *Hind*III and *Not*I digested *trp-2*_1-472_-*cA* fusion gene with *Hind*III and *Not*I digested pcDNA 3.1 His B (Invitrogen, USA). Ligated plasmid product was heat-transformed into *E. coli* TOP10 (Invitrogen, USA) cloning host.

### CHO-S cells maintenance

Suspension-adapted FreeStyle™ CHO-S cells (Invitrogen, USA) were routinely cultured in 30 ml of 8 mM L-glutamine supplemented FreeStyle™ CHO Expression Medium (Invitrogen, USA) at densities of 1.2–1.5 × 10^6^ cell/ml using disposable Erlenmeyer tissue culture flasks with vented caps (Thermo-Scientific, USA) and shaken at 125–135 rpm on orbital shakers placed in an incubator (37 °C, 8 % CO_2_). Cell viability and confluency were monitored by manual haemocytometer assisted cell counting which confirmed 98 % non-aggregating cell viability during cell maintenance under the described condition.

### Plasmid DNA transfection into CHO-S cells

Large scale plasmid extraction was carried out by using QIAGEN ® Plasmid Maxi Kit (QIAGEN, USA) in order to obtain a concentrated (~1 mg/mL) target pcDNA: *trp-2*_1-472_-*cA* expression plasmid for transfection. Cells were sub-cultured with fresh medium at 0.5–0.6 × 10^6^ cells/ml density a day prior to transfection, in order to obtain 1–1.5 × 10^6^ cells/ml confluency for transfection. The cell density was adjusted at 1 × 10^6^cells/ml and the culture medium was replaced with fresh culture medium. A total 480 ml culture was transfected by separately transfecting 16 replicates of 30 ml cell culture in 125 ml Erlenmeyer tissue culture flasks per constructed plasmids. The transfection was carried out by diluting 37.5 μl of FreeStyle™MAX reagent (Invitrogen, USA) and 37.5 μg plasmid DNA separately into 0.6 ml of Opti-PRO™ Serum Free Medium, SFM (Invitrogen, USA), and then the two solutions weremixed together. The resulting 1.2 ml DNA-FreeStyle™MAX reagent mixture was incubated at room temperature for 10 min. The mixture was then added drop wise into 1 × 10^6^ cells/ml cell suspension while gently swirling the flask and transfected cells were subsequently shaken at 135 rpm for three days in a CO_2_ incubator at 37 °C. Cells were harvested on the third day post-transfection for protein analysis by SDS-PAGE and Western Blot.

### Protein expression

Approximately 0.3 g in wet weight of pcDNA: *trp-2*_*1-472*_*-cA* transfected CHO-S cells and wild type CHO-S cells were resuspended in 1 mL of 20 mM of sodium phosphate buffer, pH 7.5 before being sonicated respectively in ice for 5 min with 50 % pulse at maximum microctip sonication power. The cell lysate was clarified by centrifugation at 10 000 × g for 10 min in 4 °C. The supernatant was collected for both TRP-2_24-472_-cA fusion protein expression and its subsequent PNGase deglycosylation analysis via Western blot. The supernatant fraction of both transfected and wild type CHO-S cell were also used for the following TRP-2 subunit enzymatic assay.

### PNGase deglycosylation analysis

The glycosylation status of TRP-2-cA fusion protein was determined by PNGase F (New England Biolabs, USA) deglycosylation assay. Briefly, 9 μl of crude transfected CHO-S cell lysate (~2.0 mg/ml) was mixed with 1 μl of Glycoprotein Denaturing Buffer and boiled at 100 °C for 10 min. Then, 2 μl of GlycoBuffer 2 and 10 % NP40 were added to the denatured protein mixture together with 1 μl of PNGase F enzyme. Appropriate amount of distilled water was added to obtain a final reaction volume of 25 μl. The reaction tubes were incubated at 37 °C for 2 h or 12 h. The PNGase F deglycosylated crude TRP-2-cA fusion protein was analysed comparatively to the untreated control by Western Blot.

### Dopachrome tautomerization assay

The melanogenic activity of the TRP-2 subunit within the TRP-2_1-472_-cA fusion protein was tested through dopachrome tautomerization assay as described by Aroca et al.*,* [32]. The substrate, L- dopachrome was prepared by mixing 160 μl of 0.6 mM of L-DOPA (Acros, USA) in 10 mM sodium phosphate buffer, pH 6.0 with 40 μl of 4.8 mM of sodium periodate (Acros, USA) which was also dissolved in the same buffer. The mixture was then diluted with 700 μl of 10 mM sodium phosphate buffer, pH 6.0 yielding 0.1 mM of L-dopachrome. A volume of 150 μl of 0.1 mM L-dopachrome was mixed with 50 μl of transfected and wild type CHO-S cell lysates respectively by triplicate wells of ELISA plates (Nunc™ MicroWell™ 96 Well Microplates, Thermo Scientific, USA). The ELISA plate was then incubated at 30 °C. Absorbance at 475 nm for both control and experiment reaction fractions were recorded for each 5 min interval for 20 min.

### TRP-2_24-472_-cA fusion protein purification

The TRP-2_24-472_-cA fusion protein was purified by using His-tag affinity chromatography. A total of ~6.0 g in wet weight of CHO-S cells was suspended in 20 mM phosphate buffer (pH 7.5) with 1 mM PMSF. The cells suspension was divided into aliquots, and each aliquot was sonicated in ice for 5 min with 50 % pulse at maximum microctip sonication power. Once sonicated, cells aliquots were pooled together and incubated in ice for an hour. The lysate was clarified by centrifugation at 20, 000 x g for 45 min and the resulting supernatant was filter sterilized. An appropriate amount of glycerol was added to the crude supernatant which ultimately resulted in ~55 ml of 20 mM sodium phosphate buffer (pH 7.5) with 5 % glycerol (Buffer A) crude supernatant suspension.

Affinity chromatography was conducted by using 1 ml HisTrap HP (GE Healthcare, USA) for Ni-NTA His tag purification at pH 7.5. The column was equilibrated with Buffer A before crude protein supernatant was applied to the column. The column was then washed using washing buffers; 20 mM sodium phosphate buffer pH 7.5 with 40 mM of imidazole. The bound fraction was eluted with 20 mM phosphate buffer pH 7.5 with 250 mM imidazole.

### Protein anchoring

Protein anchoring to the *L.lactis* cell wall was conducted similar to the previously described *in-trans* surface display report [31]. Overnight *L.lactis* MG1363 culture grown in M17 broth [33] was subcultured into fresh M17 broth and grown until cell density OD_600,_ reaches 0.5 or 0.6. A volume of 100 μl of cells from this suspension was harvested and resuspended in 100 μl of 1 × PBS. Approximately 30 μg of target or control proteins and 300 μl of M17 broth were added to the cell mixture. The cell-protein suspension was incubated at 30 °C with gentle shaking (70–90 rpm) for 2 h. The cell-protein suspension was centrifuged and the cell pellet washed thrice with 1 × PBS. The final washed cell pellet was resuspended in 200 μl of 1 × PBS for immunofluorescence microscopy analysis.

### Immunofluorescence microscopy analysis

Cell wall anchoring status was determined by immunofluorescence microscopy based on fluorescence conjugated secondary antibody tagging. The Teflon Coated Immunofluorescence Slide (Polysciences, USA) 6 mm well was treated with 0.01 % of Poly-L-Lysine solution (Sigma-Aldrich, USA) and washed thrice with 1 × PBS. A volume of 50 μl *L.lactis* cells (~1 × 10^4^ cells) was fixed on the slide well and incubated at room temperature for 30 min followed by 1 × PBS washing. Cells were then coated with 4 % paraformaldehyde and incubated at room temperature for 20 min before being washed three times with 1 × PBS. The cells were blocked for 30 min with 3 % BSA blocking solution and washed three times with 1 × PBS. The fixed cells were incubated with Anti-TRP2 Rabbit Polyclonal Antibody ab74073 (Abcam, USA) (0.2 μg/μl) in 1 % BSA (dissolved in 1 × PBS), for 1 h at room temperature. The cells were then washed thrice with 1 × PBS and incubated for 1 h at room temperature with Alexa Flour ® 488 goat anti-rabbit Antibody (Molecular Probes ®, USA) (2 μg/μl) prepared by 1:200 dilution in 1 % BSA (dissolved in 1 × PBS). Cells were subsequently washed thrice with 1 × PBS and subjected to 4’,6-Diamidino-2-phenylindole, DAPI nucleic acid staining by incubating with 0.5 μg/ml DAPI, Dihydrochloride (Calbiochem, USA) for 30 min. Post 1 × PBS washing, cells were then coated with 10 μl of Flourguard Antifade Mounting Solution (Sigma-Aldrich, USA).

The method was also repeated with cells which interacted with cA protein (obtained from Microbial Biotechnology Laboratory, Faculty of Biotechnology and Biomolecular Science, UPM) as positive control using Anti His∙Tag® Monoclonal (Novagen, USA) primary antibody and Goat Anti-Mouse Flourescein Conjugated (Calbiochem, USA) secondary antibody. For a negative control, cells were mixed with 30 μg of wild type CHO-S cell crude intracellular protein in 1 × PBS using the same method described earlier.

## Results

### Plasmid construction

**Fig. 1:**
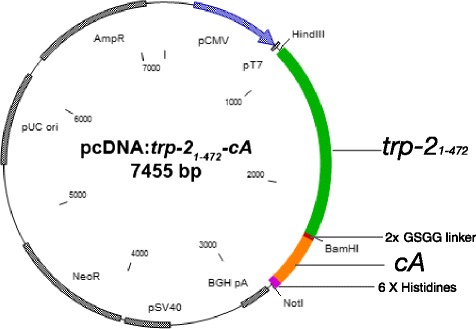
Schematic diagram of pcDNA:*trp-2*_*1-472*_ *-cA*

In this study the mammalian expression plasmid harbouring a *trp*_1-472_*-cA* fusion gene, pcDNA: *trp*_1-472_*-cA* was constructed. The heterologous gene was codon optimised, synthesized and cloned into pcDNA 3.1 His B (Invitrogen, USA). The native murine TRP-2 signal peptide was replaced with the Chinese Hamster TRP-2 signal peptide as well as six histidine repeats and an enterokinase site was included at the 3’-end of the fusion gene. These modifications were carried out to aid target glycoprotein production in CHO-S cells and its subsequent purification. The resulting plasmid and heterologous fusion gene is described in Fig. 1.

### Production & purification of TRP-2_24-472_-cA fusion glycoprotein

**Fig. 2:**
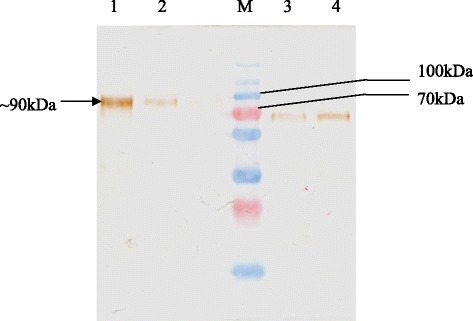
Deglycosylation of TRP-2_24-472_-cA fusion protein by PNGase treatment. Lane M: PageRuler™ Prestained Plus Protein Ladder (Fermentas, Canada); Lane 1 : Undigested TRP-2_24-472_-cA fusion protein; Lane 2: Replicate of undigested TRP-2_24-472_ -cA fusion protein Lane 3: PNGase digested TRP-2_24-472_-cA fusion protein; Lane 4: Replicate of PNGase digested TRP-2_24-472_-cA fusion protein

The production of TRP-2_24-472_-cA fusion glycoprotein by pcDNA: *trp*_1-472_*-cA* transfected CHO-S cells was analysed by SDS-PAGE and Western Blotting. Based on the Western Blot, the harvested crude protein supernatant showed TRP-2_24-472_-cA as a specific band detected at ~ 90 kDa (Fig. 2). Since the predicted molecular weight with the absence of glycosylation for TRP-2_24-472_-cA fusion protein was ~ 74 kDa, the detection of a specific band with an excess of ~16 kDa was a preliminary indicator of potential glycan moieties present in the target protein. This was confirmed by PNGase F digestion which resulted in significant reduction in TRP-2_24-472_-cA protein molecular weight from ~90 kDa to ~70 kDa after digestion (Fig. 2). Subsequently, the bio-functionality of the TRP-2 subunit in the target fusion protein was analysed through its ability to catalyse dopachrome tautomerization. The distinct decolorization of the L-dopachrome substrate (reduction in A_475 nm_) was observed after 20 min incubation at 30 °C in the crude protein fraction containing TRP-2_24-472_-cA fusion protein-substrate mixture, in contrast to wild-type CHO-S cell crude protein-substrate mixture (Table 1). This indicates that the TRP-2 subunit in fusion glycoprotein is enzymatically active.

**Table 1:** OD475nm absorbance reading of L-dopachrome respectively mixed with wild-type CHO-S and transfected CHO-S crude protein

Sample	Wild type CHO-S crude protein extract			pcDNA: *trp-2*_*1-472*_ *-cA* transfected CHO-S cell crude protein extract		
Replicates	1	2	3	1	2	3
0 min	0.203	0.205	0.202	0.205	0.208	0.206
5 mins	0.196	0.199	0.194	0.173	0.179	0.175
10 mins	0.184	0.188	0.180	0.140	0.147	0.143
15 mins	0.179	0.182	0.176	0.101	0.106	0.104
20 mins	0.170	0.173	0.168	0.093	0.098	0.095

**Fig. 3:**
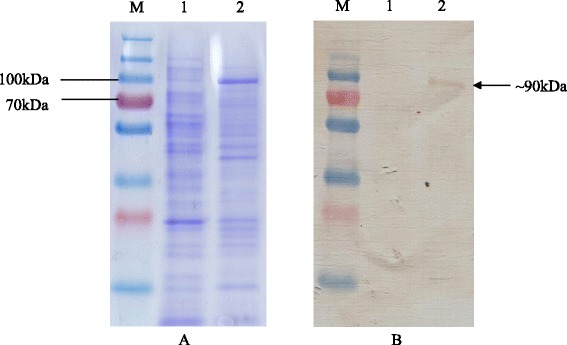
Purification of TRP-2_24-472_-cA by His-tag affinity chromatography. **a** SDS-PAGE profile; (**b**) Anti-His Western Blot profile. Lane M: PageRuler™ Prestained Plus Protein Ladder (Fermentas, Canada); Lane 1: His-tag affinity chromatography unbound fraction; Lane 2: His-tag affinity chromatography 250 mM imidazole eluted fraction

The crude supernatant harvested from ~6.0 g of cells (wet weight) was subjected to His- tag affinity chromatography. Western blot analysis of the eluted fraction resulted in ~90 kDa target protein eluted at 250 mM imidazole (Fig. 3).

### Immunofluorescence microscopy analysis

**Fig. 4:**
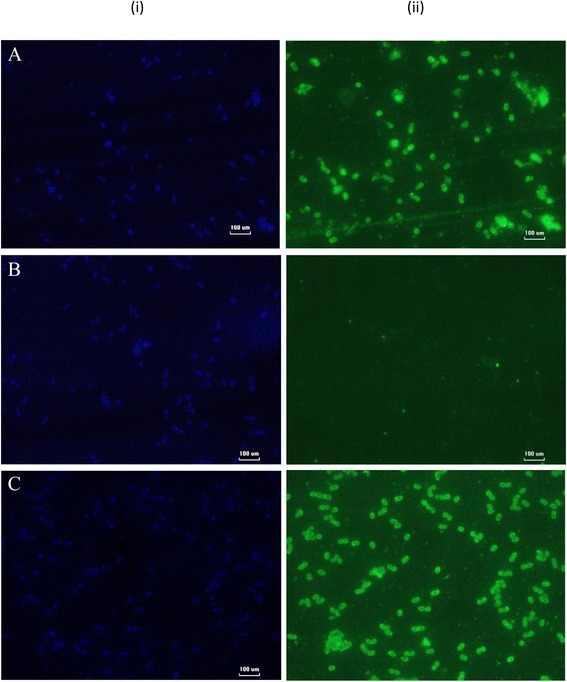
Immunoflouroscence microscopy of *L.lactis* interacted with the TRP-2_24-472_-cA glycoprotein. (i) DAPI nucleus staining for *L. lactis* cells (ii) FITC conjugated Secondary Antibody Staining. **a** *L. lactis* interacted with cA protein (expressed and purified *E.coli*), (**b**) *L. lactis* interacted with wild type CHO-S cells crude intracellular protein in 1 x PBS, (**c**) *L. lactis* interacted with TRP-2_24-472_-cA glycoprotein

Cell wall anchoring of glycosylated target protein, TRP-2_24-472_-cA was analysed to determine the bio-functionality of cA, the cell wall anchoring LysM motif. It was observed that the *L. lactis* MG1363 cells treated with TRP-2_24-472_-cA and cA protein alone (positive control for immunofluorescence microscopy) respectively, emitted green fluorescence signals in contrast to those treated with the negative control (*L.lactis* treated with wild-type CHO-S crude protein in 1 × PBS) (Fig. 4). This confirmed that the target glycoprotein, TRP-2_24-472_-cA, was anchored to the cell wall of *L. lactis* MG1363. In order to determine if the location of the target glycoprotein correlated with the presence of the *L. lactis*, DAPI staining was performed. As shown in Fig. 4, TRP-2_24-472_-cA glycoprotein co-localized with *L. lactis*. Therefore, the target TRP-2_24-472_-cA glycoprotein was expressed *in-trans* by *L. lactis* cells through the successful anchoring of the target to its cell wall.

## Discussion

In this work, an *in-trans* surface display system was utilized to generate *L. lactis* surface displaying the mammalian glycoprotein TRP-2. A vaccine using non-recombinant prokaryotes to deliver glycosylated eukaryotic proteins, is an attractive one, especially due to the low cost involved in generating sufficient quantities for the delivery system. Hitherto, bacterial *in-trans* and recombinant surface display of heterologous proteins were very much associated with non-glycosylated protein produced by prokaryotic cell factories. Such an approach is not suitable when the target protein requires post-translational modifications for protein folding and bio-functionality.

*L. lactis* surface display applications have been predominantly involved in antigen or epitopes delivery for mucosal immunization through the oral route. This concept was first demonstrated in the streptococcal system by fusing the E7 protein of human papillomavirus type 16 with the C-terminal attachment motif of fibrillar M6 protein from *Streptococcus pyogenes* [34]. Since then, a similar strategy has been extensively manipulated in the lactococcal system by initially using sortase mediated cell wall anchoring motifs such as, LPXTG casein serine protein from *L. lactis* PrtP protein [35], *S. pyogenes* M6 protein [36] and *Staphylococcus aureus* protein A (SPA) anchor [37]. The non-covalent cell wall binding domain, cA (LysM-AcmA) domain, was compared with LPXTG motifs, i.e. PrtP using recombinant technology in surface displaying F18 Fimbrial Adhesin FedF, and was concluded to be more efficient [28].

Despite successful heterologous protein surface display for vaccine delivery by *L.lactis* through the nisin controlled gene expression system (NICE®), genetic modification of bacteria has been a safety concern. The GRAS status of *L. lactis* and many other LABs is insignificant as genetically modified microorganisms pose a risk of horizontal gene transfer, particularly antibiotic resistance gene transfer, in human and animal applications [10]. These challenges can be overcome by the *in-trans* surface display strategy which involves external cell wall binding of target protein –LysM (AcmA) fusion proteins produced from a host separate from the protein carrier. Such fusion proteins have been successfully produced in *L. lactis* [38], *E.coli* [9] and *Pichia pastoris* [39] before being purified and anchored to LAB peptidoglycan*.* The AcmA cell wall anchor also been used to bind recombinant proteins to killed *L. lactis* termed Gram-positive Enhancer Matrix (GEM) cells [40] as this system increases protein binding by removing hindering cell wall compartments and improves the biosafety of non-recombinant *L. lactis* vaccine.

Recently, N-glycosylation has been identified to significantly enhance epitope presentation of MHC class I molecules as demonstrated by using tyrosinase as a model antigen [41]. This is due to de-glycosylation by peptide N-glycanase (PNGase) during the glycosylation process in the ER which allows deamidation for epitope presentation. Apart from that, proteolysis for epitope presentation was also discovered to slow down near the N-glycan site thus resulting in the generation of native epitopes [42]. Previously however, the surface display strategy for glycosylated proteins has been restricted to yeast [43], but this system compromises the quality of the target glycoprotein, since yeast attach a different linkage of carbohydrate moieties (primarily mannose) to the core glycosyl unit and the system is highly susceptible to hyperglycosylation. Due to these differences, there is a preference for utilizing mammalian cells over yeast in generating therapeutic glycoproteins [44, 45]. The present study addresses these problems by utilizing the *in-trans* surface display system for glycoprotein delivery. In this study, TRP-2 glycoprotein fused with *L. lactis* N-acetylmuramidase C-terminal LysM cell wall anchor, cA, was expressed in a mammalian cell (CHO-S cells) and subsequently anchored to the *L. lactis* cell wall. This study demonstrates that glycosylated antigens can be produced in a mammalian cell system and then can be anchored to a bacterial cell surface, by fusion with a cell wall anchoring motif such as cA.

Previously, most reported peptide based TRP-2 vaccines utilized highly antigenic short synthetic peptides (epitopes) i.e. TRP-2_180-188_ [46, 47], chiefly due to the difficulties in purifying the full length TRP-2 [32, 48]. Such an approach compromises the full length glycosylated TRP-2 immunogenic potential. Hitherto, high purity TRP-2 glycoprotein was successfully obtained from Sf9 insect cells but only upon the removal of the hydrophobic C-terminal transmembrane domain [48].

In this study, TRP-2 devoid of the transmembrane domain(TRP-2_24-472_) was utilized to generate recombinant protein. In addition, in order to promote TRP-2 glycosylation and signal peptide recognition in the CHO-S cell expression system, the murine TRP-2 signal peptide, mTRP-2sp was substituted with Chinese Hamster TRP-2 maturation signal peptide, chTRP-2sp. Despite 91 % amino acid sequence similarity between Chinese Hamster TRP-2 and murine TRP-2, the signal peptidase cleavage sequence varies considerably and the murine version was unable to promote proper localization in the CHO-S system (data not shown). Production of soluble TRP-2_24-472_-cA by CHO-S cells enabled His-tag purification which resulted in the production of “partially purified” TRP-2_24-472_-cA from recombinant CHO-S cells. Even though purity was poor, this method resulted in a protein fraction with enriched target protein.

The final phase of this study was to analyse the ability of TRP-2_24-472_-cA glycoprotein to anchor onto the *L. lactis* cell wall. This assay was first demonstrated by Steen et al.*,* [38] by interacting 15 μg of cA protein expressed and purified from *L. lactis* NZ9700 with trichloroacetic acid-treated *L. lactis*. Since then, the *in-trans* approach has been applied to externally introduced target protein expressed and purified from *E. coli* [9, 30, 31] to the *L. lactis’* cell wall. Similarly, the successful expression and purification of TRP-2_24-472_-cA fusion glycoprotein from CHO-S cells in this study generated a novel TRP-2 glycoprotein with *L. lactis* cell wall anchoring properties. The cA domain cell wall anchoring feature was confirmed by visualization of TRP-2_24-472_-cA glycoprotein mixture with live *L. lactis* through immunofluorescence microscopy (Fig. 4). The functionality of prokaryotic (*L. lactis*) cell wall anchoring domain, cA expressed and purified from eukaryotes has been previously demonstrated [39]. The aforementioned study successfully docked yeast produced cA, which was mutated to eliminate N-glycosylation sites thought to possibly hinder docking, onto the cell surface of *Lactobacillus casei* NRRL B-441 [39]. Despite none of the predicted N-glycosylation sites present in the cA domain were eliminated in this study, the anchoring motif was shown to be fully functional and capable of anchoring glycosylated TRP-2 onto the *L. lactis’* cell wall.

While the concept of surface displaying glycosylated eukaryotic proteins on prokaryotic bacteria using the above approach was proven successful, the major drawback has been the initial low expression of the target fusion protein produced by the transient CHO-S expression system. This restriction has accounted for difficulties in accumulating sufficient amount of the pure target proteins, limiting the study to the characterization of the glycosylation of the TRP-2_24-472_-cA fusion protein and immunogenicity analysis using in vivo models. Optimization of the current transient expression system or development of a stable cell line expressing the TRP-2_24-472_-cA fusion protein will have to be carried out before the system can be fully utilized.

## Conclusion

In summary, this study represents a novel method of displaying eukaryotic proteins on bacterial carriers such as *L. lactis* when post-translational glycosylation is crucial for functionality. The system developed in this study, i.e. *L. lactis* surface displaying TRP-2, is anticipated to be able to function as a cancer vaccine which will be investigated in future studies once sufficient target protein production is achieved. The vaccine which could be administered orally or intranasally, would likely to induce an antigen specific immune response against glioma and melanoma cells, triggering immune effectors including antigen specific CD8+ cells and antibodies as has been detected in other studies utilizing LAB as antigen carriers.

## Abbreviations

cA: C-terminal lysin motifs of *L. lactis* N-acetylmuramidase
CHO-S: suspension adapted Chinese Hamster Ovary cells
DAPI: 4’,6-Diamidino-2-phenylindole
FITC: fluorescein isothiocyanate
GRAS: generally regarded as safe
LAB: lactic acid bacteria
L-DOPA: L-3,4-dihydroxyphenylalanine
LysM: lysin motifs
PBS: phosphate buffer saline
PMSF: Phenylmethylsulfonyl fluoride
SAM: surface anchoring motifs
TRP-2: tyrosinase related protein 2
